# Research on the Policy Effect and Mechanism of Carbon Emission Trading on the Total Factor Productivity of Agricultural Enterprises

**DOI:** 10.3390/ijerph19137581

**Published:** 2022-06-21

**Authors:** Junguo Hua, Di Zhu, Yunfei Jia

**Affiliations:** School of Economics and Management, Henan Agricultural University, Ping An Avenue 218, Zhengzhou 450000, China; hjghnnd168@163.com (J.H.); 18203749635@163.com (D.Z.)

**Keywords:** carbon emissions trading, total factor productivity of agricultural enterprises, green innovation, double difference

## Abstract

Given the rural revitalization strategy in the new era, agricultural development is faced with the dual constraints of resources and the environment. Promoting the green development of agriculture is one of the important missions to solve major social issues in the new era. The implementation goal of the carbon emission trading system is to achieve a win-win situation between carbon emission reduction and green development. To evaluate the effectiveness of the carbon emission trading system on agricultural enterprises, this paper uses a double-difference model to analyze the policy effect and mechanism research path of the impact of the carbon emission trading system on the total factor productivity of agricultural enterprises. The results based on the panel data of listed agricultural companies from 2010 to 2020 show that (1) carbon emission trading rights have significantly improved the total factor productivity of agricultural enterprises; (2) green innovation in carbon emission trading rights have an impact on the total factor productivity of agricultural enterprises; and (3) heterogeneity analysis shows that the effect of carbon emission trading rights on the total factor productivity of agricultural enterprises mainly exists in large-scale, nonstate-owned, high-debt enterprises, enterprises in the eastern region, and enterprises with government subsidies. Therefore, in the future, China should continue to implement the current carbon emission trading rights system in air pollution control, and at the same time, it needs to be supplemented by government intervention and other means for long-term governance. In conclusion, the study provides a reference value for promoting the realization of the long-term goal of “low carbon” and “high quality” green development of agricultural economy and for making reasonable and effective behavioral decisions for the survival and development of enterprises.

## 1. Introduction

The frequent occurrence of extreme weather has made the problem of environmental pollution an important issue in countries around the world. To protect the natural environment and alleviate air pollution, the European Union Emissions Trading System (EUETS) came into being in the early 20th century and developed rapidly as an important driver for the high-quality development of enterprises. According to the research data of the World Bank, the carbon emission trading policies reduced carbon emissions by 2–5% during the period of 2005–2007, with an annual reduction of 40–100 million tons of carbon emissions. This shows that the carbon emission trading policies are of great significance in reducing emissions and promoting the development of global green technology applications. Since the implementation of the reform and opening-up policy, China’s carbon emissions have continued to grow. Especially after China’s entry into the WTO, with the advantages of resources and demographic dividends and the “4 trillion” plan in 2008, the scale of foreign trade has continued to increase, the manufacturing industry has expanded rapidly, and carbon dioxide emissions have increased. China’s carbon dioxide emissions account for nearly 30% of global emissions. From 2002 to 2007, the average growth rate of carbon emissions reached 14%, making China gradually become the world’s largest energy consumer and carbon dioxide emitter. As a responsible major country, China has realized the importance of harmonious coexistence between economic development and the natural environment, and has begun to emphasize “green development”. The five development concepts of “innovation, coordination, greenness, openness, and sharing” put forward by the Fifth Plenary Session of the 18th Central Committee have elicited a top-to-bottom consensus at the national level. In response to global warming, the Chinese government has adopted a series of policy measures. In 2011, the National Development and Reform Commission issued the “Notice on Carrying out the Pilot Work of Carbon Emissions Trading”, formally approving pilot work on carbon emissions trading in Beijing, Shanghai, Shenzhen, Guangdong, Tianjin, Hubei, and Chongqing. In 2012, carbon emission trading markets were established in Beijing, Shanghai, and other regions. In December 2017, the construction of the national carbon emission trading market was launched. China has entered the era of carbon emissions trading [1]. The Fifth Plenary Session of the 19th Central Committee of the Communist Party of China once again emphasized that the shift of economic growth to the mode supported by the promotion of total factor productivity of enterprises is the inevitable choice for China’s economy to face the decline of capital returns and the disappearance of demographic dividends and the source of power for achieving high-quality economic development [2]. Agriculture is a barometer of the national economy; listed agricultural companies are the leaders of the agricultural industry, shouldering the important mission to drive farmers to increase production and income, promote agricultural industrial structure adjustment, and promote the process of agricultural industrialization. Their management level represents the reality of China’s agricultural industry and development prospects. Therefore, it is of great significance to study the impact of the carbon trading pilot policies implemented in China on the total factor productivity of listed agricultural enterprises under the actual market environment and institutional conditions. The carbon emission trading policy mainly focuses on regulating carbon emission reduction at the enterprise level. Agricultural enterprises regulated by carbon emission trading may respond by accelerating green innovation from the perspective of compliance cost and innovation compensation [3], thereby indirectly affecting the full factor production rates of enterprises. Therefore, effectively quantifying the impact of carbon emission trading policies on agricultural enterprises’ total factor productivity and its internal mechanism is helpful for the study of improving enterprises’ total factor productivity against the background of “carbon peaking” and “carbon neutrality” and further promoting the quality of economic growth, the coordinated promotion of high-quality economic development in China, and the implementation of new development concepts play a pivotal role.

Taking listed agricultural companies from 2010 to 2020 as samples, this paper uses endogenous growth theory and cost and innovation compensation theory as the basis, with the help of logical reasoning and mathematical derivation, to analyze the impact of carbon emission trading rights on the total factor productivity of agricultural enterprises and explore its mechanism. On this basis, an empirical test is carried out, and the effect of heterogeneity is further analyzed. The possible marginal contributions of this paper are as follows: First, this paper makes an accurate assessment of the environmental and economic effects of the carbon emission trading pilot policy to provide direct empirical evidence for the further improvement of the carbon emission trading policy, which is a useful supplement to the empirical research field of carbon trading in China. Second, from the perspective of microagricultural enterprises, this paper examines the impact of emission trading policies on the total factor productivity of listed agricultural enterprises and the possible emission reduction mechanism and expands the research perspective in related fields to provide a reference for agricultural enterprises to meet the current “low carbon” and “high quality” economic development requirements. Third, this paper provides new evidence and explanations for the use of market-oriented environmental regulation to improve the total factor productivity of listed agricultural companies, thereby promoting the high-quality transformation of China’s agricultural economy, and enriches the literature on the economic effects of carbon emission trading policies from a micro perspective. Fourth, the evidence and findings obtained from this study can provide a supportive experience and policy suggestions for China to improve the carbon trading mechanism and implement a unified national carbon trading market.

## 2. Literature Review

Domestic and foreign studies on the impact of environmental regulation on total factor productivity have not reached a consistent conclusion. “Follow the cost” theorists believe that the cost of environmental regulation inhibits the improvement of enterprise productivity and international competitiveness, resulting in a decline in the total factor productivity of enterprises [4,5,6]. In contrast, the “innovation compensation” theorists believe that the public nature of environmental protection determines the inefficiency of the market mechanism in its allocation. Enterprises will not take the initiative to carry out technological innovation, but environmental regulation can enable enterprises to internalize external costs and stimulate them. Enterprises carry out innovation and promote the improvement of the total factor productivity of enterprises [7,8,9,10]. At the same time, some scholars believe that the impact of environmental regulation on total factor productivity depends on the relative magnitude of the above two effects, so they pay more attention to the nonlinear relationship between environmental regulation and total factor productivity. The effectiveness and cross-effect of green innovation were re-examined [11,12], while others pointed out that there were regional differences in the promotion of environmental regulation and technological innovation, and there was a “U”-shaped relationship between the two [13]. Liu Siming believes that the relationship between environmental regulation and productivity growth is nonlinear or uncertain, and the relationship between the two will vary due to different industries and types of environmental regulation [14]. Although there are abundant studies on the impact of environmental regulation on total factor productivity, few scholars have explored it from the perspective of agricultural enterprises.

The types of environmental regulation in China can be roughly divided into three types: command-and-control, market-incentivized, and public participation. Previous studies have investigated the command-and-control environmental regulation represented by the “two-control zone”, which increases the production cost of regulated enterprises and leads to a decrease in the total factor productivity of enterprises. Market-incentivized environmental regulation, represented by sulfur dioxide emission rights, improves the total factor productivity of enterprises by promoting enterprise innovation [15]. Public participation in environmental regulation represented by environmental information disclosure can also promote the total factor productivity of enterprises [16]. However, there are relatively few studies examining the impact of carbon emissions trading, one of China’s current important carbon emission reduction policies, on total factor productivity. In addition, related studies on the impact of carbon emission rights trading have also given more attention to carbon intensity, carbon emissions [17], carbon allocation [18], enterprise value [19], enterprise innovation [20], enterprise green innovation [21], and low-carbon technology collaborative sharing against the background of carbon emissions trading [22], and less research on the impact on agricultural enterprises’ total factor productivity has been conducted.

The main goal of traditional technological innovation is to bring economic benefits to enterprises, while green technological innovation, as an important part of traditional technological innovation, emphasizes the comprehensive benefits of the economy, environment and society. In recent years, with the continuous evolution of the needs of economic and social development, green technology has been frequently applied in the fields of energy conservation and environmental protection, clean energy, cleaner production, and the circular economy [23]. Due to the characteristics of the “dual externalities” of green technology innovation, in the absence of market pricing mechanism constraints and policy interventions, there will be insufficient motivation for enterprises to innovate. As an important market-based environmental policy, carbon emission trading has a reasonable price formation mechanism and government management mechanism, which can eliminate “double externalities” to a large extent and encourage enterprises to carry out green technology innovation. As the best way to solve the contradiction between the environment and development, technological innovation requires enterprises to pay more attention to ecological and environmental factors when solving the above problems [24]. Therefore, it is of great significance to explore the impact of green technology innovation on the total factor productivity of agricultural enterprises under the carbon emission mechanism.

By sorting out the relevant literature, it is not difficult to see that there are few studies evaluating the environmental and economic effects of carbon emission trading in China. From the perspective of microagricultural enterprises, this paper uses the double-difference method to study and analyze the data of listed agricultural companies from 2010 to 2020, focusing on the impact of the total factor productivity of listed agricultural companies after the implementation of the carbon emission trading system. Introducing variables of green technology innovation can better explain the impact of the carbon emission trading system on enterprise productivity and its influential mechanism. The study conclusions provide a basis for the government to establish a national carbon emission trading market for the evaluation of the economic effects of carbon emission rights and have great practical significance for making reasonable and effective behavioral decisions for the survival and development of enterprises.

## 3. Materials and Methods

### 3.1. Theoretical Mechanism of Carbon Emission Trading Rights and Total Factor Productivity of Agricultural Enterprises

Carbon emissions trading internalizes the external cost of carbon emissions of agricultural enterprises through the price mechanism to reduce the carbon dioxide emissions of enterprises [25]. The direct effect of it has two aspects. One is the cost-push effect. To achieve the emission reduction target, the Chinese government sets a strict total carbon emission amount according to social needs and then decomposes the total carbon emission target layer by layer to each lower level. In this way, carbon emission rights are commercialized. After agricultural enterprises are constrained by the carbon emission rights trading mechanism, if they want to discharge more than the quota, they need to purchase from the government or the market, which will weaken the technological innovation ability of enterprises and reduce the overall efficiency of the factor productivity of agricultural enterprises [26], otherwise it will be punished accordingly, which will undoubtedly increase the extra cost of relevant subjects, thus forcing each subject to use carbon emission allowances efficiently. The second is the income incentive effect. By designing a system that meets the environmental conditions of the Chinese market, carbon emission rights are allowed to be traded under certain rules, and relevant entities gain more choice space and can choose to buy or sell under their own cost constraints without quota. When the carbon price in the trading market is higher than the emission reduction cost, the relevant entities can have excess emission allowances through emission reduction and sell the allowances in the carbon emission rights trading market; when the carbon price in the trading market is lower than the emission reduction cost, the relevant entities can buy carbon emission allowances in the market through carbon emission trading. Carbon trading can form an effective allocation of the market so that enterprises with lower emission reduction costs can reduce carbon emissions more, and enterprises with higher emission reduction costs can reduce carbon emissions less so that the total cost of emission reduction in society can reach the lowest value. Therefore, agricultural enterprises have incentives to benefit from carbon emission trading by selling multiple carbon emission quotas and improving the competitiveness of enterprises. At the same time, higher productivity is generated by innovation incentives, efficiency improvement, and redistribution [27]. Based on this, this paper proposes Hypothesis 1 for the emission reduction effect of carbon emission trading policies:

**Hypothesis** **1** **(H1).** *The pilot policy of carbon emission trading promotes the improvement of the total factor productivity of agricultural enterprises*.

### 3.2. Theoretical Mechanism of Green Innovation in Carbon Emission Trading Rights and Total Factor Productivity of Agricultural Enterprises

If China’s carbon emission trading pilot policy can effectively improve the total factor productivity of agricultural enterprises, how does it achieve this effect? On the one hand, under the conditions of a given carbon quota, for enterprises in the carbon emission pilot areas, the pilot policy will increase the carbon emission cost of enterprises in the pilot areas, thereby increasing the operational burden of enterprises. Enterprises actively carry out technology research and development, which can reduce carbon emissions, thereby reducing the pressure on corporate emissions. On the other hand, companies carry out green technology innovations to reduce their carbon emissions to obtain excess carbon emission credits. These excess carbon emission credits can generate profit for companies selling them in the carbon emissions trading market. In the long run, when profit-seeking enterprises face the constraints of carbon emission limits, realizing low-carbon production through green innovation is the optimal choice for enterprises. Therefore, carbon trading policies force companies to carry out green innovation activities and reduce carbon emissions per unit of output. This approach can not only make the total carbon emissions of the enterprise meet the established carbon emission constraint target but also make a profit by selling the saved carbon emission credit in the carbon emission rights trading market. The technological innovation capability of an enterprise is an important factor affecting the total factor productivity of an enterprise. To this end, this paper selects the green innovation effect path to test the transmission mechanism. To investigate whether the pilot policy of carbon emissions trading can improve the total factor productivity of enterprises in the pilot area by improving the level of green innovation of enterprises, this paper proposes Hypothesis 2:

**Hypothesis** **2** **(H2).** *The pilot policy of carbon emissions trading improves the total factor productivity of enterprises by promoting enterprise innovation*.

## 4. Research Design

### 4.1. Sample Selection and Data Sources

To test the above assumptions, according to the CSRC industry classification standard, this paper selects all A shares listed on the Shanghai and Shenzhen Stock Exchanges and GEM belonging to agriculture; forestry; animal husbandry and fishery; agricultural and sideline food processing; food manufacturing and wine, beverage, and refined tea manufacturing enterprises as the research object. The annual data of enterprises during the sample period from 2010 to 2020 are selected, and the relationship between carbon emission trading rights, green innovation, and total factor productivity of agricultural enterprises is analyzed. Before using the data, we processed the data as follows: (1) ST and *ST-listed agricultural companies were removed; (2) listed agricultural companies with missing main financial variables and listed agricultural companies with abnormal and missing data were removed; (3) all nonvirtual variable values were processed by 1% pre- and post-tailing to eliminate the influence of extreme values. Finally, 1340 observations were obtained. The financial data of listed companies are from the China Stock Market Accounting Research (CSMAR) Database, and the patent data of green technology of enterprises are from the National Patent Database. The total factor productivity data of agricultural enterprises are from the CSMAR and Rethink Databases, assisting Tonghuashun and Sina Finance and Economics (CSMAR: http://cndata1.csmar.com/ (accessed on 1 January 2022); National Patent Database: https://www.cnpat.com.cn/ (accessed on 1 January 2022); Rethink Databases: http://www.resset.cn/db (accessed on 1 January 2022); Tonghuashun: https://www.10jqka.com.cn/ (accessed on 1 January 2022); Sina Finance and Economics: https://finance.sina.com.cn/ (accessed on 1 January 2022)).

### 4.2. Variable Definition and Interpretation

#### 4.2.1. Explained Variables

This paper refers to the research of Li Dandan and Li DoudouLu [28] and Czyzewski et al. [29]. Considering the endogeneity problems in sample selection and statistical methods, this paper uses semiparametric methods (OP method and LP method, etc.) to calculate total factor productivity data at the firm level. First, the calculation results of the LP method are used for benchmark regression, and then the calculation results of the OP method are used for robustness testing. The calculation TFP method and index selection of this calculation method are as follows:

$$TFP_{ijt}=\alpha_{jt}+\beta_{jt}L_{ijt}+\gamma_{jt}K_{ijt}+\delta_{jt}M_{ijt}+\varepsilon_{ijt}$$

In the above formula, $TFP_{ijt}$, $L_{ijt}$, $K_{ijt}$, and $M_{ijt}$ are the t logarithms of total factor productivity, labor input, capital input, and intermediate input of private enterprises in industry i, respectively; j and $\varepsilon_{ijt}$ are random disturbance terms. The above variables are all based on 2010.

#### 4.2.2. Mechanism Variables

Corporate green innovation. There are three types of patents in China: invention, utility model, and appearance, of which technology-related patents are mainly inventions and utility models. Referring to the research of Liu Jiamin et al., the green innovation of enterprises is measured by the number of green patent applications [30]. The data on the green innovation of listed companies come from the National Patent and Property Office. The data are cleaned and screened by using the classification of green technology patents defined by the World Intellectual Property Organization (WIPO), and the total number of green patents, the number of green invention patents, and the number of green utility model patents applied for by listed companies each year are obtained. The larger the value is, the higher the level of green innovation.

#### 4.2.3. Control Variables

To reduce the estimation bias caused by omitted variables, this paper draws on the factors that may affect the total factor productivity of agricultural enterprises in the literature and selects the following variables as control variables: enterprise size (Size), return on assets (Roa), asset-liability ratio (Lev), agency cost (Agencost), cash flow from operating activities (Cflow), factor intensity (Capital), and the shareholding ratio of the largest shareholder (Top1). The specific definition of each variable is shown in Table 1.

### 4.3. Model Establishment

The impact of carbon emission rights on the total factor productivity of agricultural enterprises in the double-difference model constructed in this paper is as follows:

(1) $$TFP_{it}=\alpha_{0}+\alpha_{1}Treat_{i}*Time_{t}+\alpha_{2}C\mathrm{on}trols_{it}+\varepsilon_{i}+\varepsilon_{t}+\varepsilon_{it}$$

To test for the existence of a mechanism effect, the following model was constructed:

(2) $$TFP_{it}=\gamma_{0}+\gamma_{1}Treat_{i}*Time_{t}*Patent_{it}+\gamma_{2}Treat_{i}*Time_{t}+\gamma_{3}Patent_{it}+\gamma_{4}C{\mathrm{ontrols}}_{it}+\varepsilon_{i}+\varepsilon_{t}+\varepsilon_{it}$$

In Equations (1) and (2), $TFP_{it}$ reflects the total factor productivity i of agricultural enterprises of sample companies $\alpha_{0}$ during the observation period, where t is a constant term, $Treat_{t}$ represents a dummy variable for policy treatment (1 for provinces located in pilot areas, 0 for provinces located in nonpilot areas), and $Time_{t}$ represents the time dummy variable (1 after the policy, 0 before the policy), which is the $Treat_{i}*Time_{t}$ cross term of the treatment effect, that is, the core explanatory variable of this paper, and its estimated coefficient reflects the impact of carbon emission rights and the agricultural enterprises not affected by carbon emission rights. Regarding the average difference in total factor productivity, if $\alpha_{1}>0$, it shows that the total factor productivity of agricultural enterprises affected by carbon emission rights has a significant improvement compared with those not affected by carbon emission rights; that is, the carbon emission rights policy promotes the improvement in total factor productivity of agricultural enterprises. Patent is the mediating variable, $Controls_{it}$ is the control variable, $\varepsilon_{i}$ is the individual fixed effect, $\varepsilon_{t}$ is the year fixed effect, and $\varepsilon_{it}$ is the random disturbance term.

First, Model (1) is regressed to test the impact of carbon emission rights on the total factor productivity of agricultural enterprises. If the coefficient is significantly positive, it means that carbon emission rights have significantly improved the total factor productivity of agricultural enterprises. Then, we regress Model (2). Whether the green innovation of enterprises has played a mechanistic role in the impact of carbon emission rights on the total factor productivity of agricultural enterprises is tested. If the coefficient of the interaction term is significantly positive, it means that the green innovation of enterprises has played a mechanistic role.

## 5. Empirical Analysis

### 5.1. Descriptive Statistics

Table 2 shows the descriptive statistical results of the main variables. The average value of the total factor productivity (TFP) of agricultural enterprises is 9.081, the minimum value is 6.733, the maximum value is 11.670, and the standard deviation is 1.016, indicating that on the whole, the total factor productivity of private enterprises fluctuates greatly, and the data have a large degree of dispersion. The average value of enterprise green innovation is 1.726, and the standard deviation is 4.519. The number of green patent applications of enterprises varies greatly.

### 5.2. Analysis of Regression Results

The Hausman test rejects the null hypothesis. Therefore, this paper uses a fixed-effect double-difference model to explore the impact of the carbon emission trading pilot policy on the total factor productivity of agricultural enterprises. At the same time, the reliability of the estimated results is tested by adding control variables. The regression results of Model (1) are shown in Table 3. Column (3) shows the regression result of the double-difference model, and Columns (1) and (2) show the regression results of the double-difference model after controlling for individual and year-fixed effects.

The regression results in Column (1) of Table 3 show that the coefficient of the interaction term is significantly positive at the 1% level; compared with the control group, the carbon emission trading pilot will increase the total factor productivity of agricultural enterprises in the treatment group, and the carbon emission trading pilot policy will increase the total factor productivity of agricultural enterprises by about 0.316% on average. In Column (2), control variables are added on the basis of Column (1), and the regression results are still significantly positive. After considering the influence of other factors, the pilot carbon emissions trading policy increased the total factor productivity of agricultural enterprises by about 0.149% on average; R2 also increased significantly, indicating that after adding control variables, the model fit was better, and the research conclusions were still robust, while Column (3) controlled the individual and time fixed effects on the basis of Column (2). R2 in Column (3) increased significantly, indicating that there are indeed differences at the individual and time levels that can affect the total factor productivity of agricultural enterprises. Therefore, it is reasonable to add individual and time-fixed effects to the benchmark regression in this paper. Regardless of which method is used for regression, the coefficient of the interaction term of the explanatory variables is significantly positive, indicating that the pilot policy of carbon emission trading can improve the total factor productivity of agricultural enterprises to a certain extent. Hypothesis 1 was preliminarily verified. To make the research conclusions more credible, this paper conducts a series of robustness tests. In addition, the R2 of the model in Table 3 is greater than 0.1, which is acceptable considering the complexity of the research phenomenon.

### 5.3. Robustness Test

#### 5.3.1. Parallel Trend Test

Satisfying the parallel trend assumption of the experimental group and the control group is one of the basic prerequisites for using the double-difference method. Therefore, before the carbon trading pilot (2013), the evolution of the indicators in the experimental group and the reference group should remain basically the same and consistent; otherwise, the regression results may be biased. This paper refers to the practice of predecessors and uses the dummy variable Pre4~After5 to replace (1) in the formula. The $Treat_{}*Time$ specific regression equation is as follows:

(3) $$\begin{array}{l} TFP_{it}=\alpha_{1}+\alpha_{-4}{\mathrm{Pre}4}_{it}+\alpha_{-3}{\mathrm{Pre}3}_{it}+\alpha_{-2}{\mathrm{Pre}2}_{it}+\alpha_{-1}{\mathrm{Pre}1}_{it}+\alpha_{0}{\mathrm{Current}}_{it}+\alpha_{1}{}^{\prime }\mathrm{After}1_{it}\\ +\alpha_{2}{}^{\prime }{\mathrm{After}2}_{it}+\alpha_{3}{}^{\prime }\mathrm{After}3_{it}+\alpha_{4}{}^{\prime }{\mathrm{After}4}_{it}+\alpha_{5}{}^{\prime }\mathrm{After}5_{it}+\alpha_{2}\mathrm{Controls}+\varepsilon_{it} \end{array}\;$$

In Equation (3), $\mathrm{Pr}e4_{\mathrm{it}}$ means that t in the fourth year i before the enterprise is affected by the carbon trading pilot, when the enterprise is in the fourth year before being affected by it, the variable takes a value of 1; otherwise, it takes a value of 0. $After1_{it}$ means the t in the first year i after the enterprise is affected by the carbon trading pilot; when the enterprise is in the first year after being affected by it, the variable takes the value 1; otherwise, it takes a value of 0. $Current_{it}$ means that t in the current period when the enterprise is affected by the carbon trading pilot, when the enterprise is in the current period i affected by it, the variable takes a value of 1; otherwise, it takes a value of 0. The definitions of the remaining variables are the same as the regression results, and from the test results in Column (1) of Table 4, it can be seen that the regression coefficients of Pre3~Current are not significant, and the regression coefficients of After1~After6 are all significant. The above results show that before the arrival of the carbon trading pilot, there was no significant difference between the experimental group and the control group, which satisfies the parallel trend hypothesis. The carbon trading pilot has a good effect in promoting the total factor productivity of agricultural enterprises, but the implementation of the policy has a certain lag. On the whole, the research design of this paper conforms to the premise of using the double-difference method.

#### 5.3.2. Parallel Trend Test

To ensure the robustness of the experimental results, this paper draws on the practice of Topalova [31] and uses the idea of a counterfactual test to conduct a placebo test. Specifically, we set the sample data before the carbon trading pilot, assuming that the carbon trading pilot occurred in 2012 and 2011 and conducted regression analysis again. If the increase in the total factor productivity of agricultural enterprises was indeed caused by the carbon trading pilot in 2013, the coefficient of the interaction term should be insignificant in the regression results of the fictitious carbon trading pilot year. Columns (2) and (3) of Table 4 report the test results, and the coefficients of the interaction terms are 0.067 and 0.159, but not significant. This means that the fictitious policies have increased the total factor productivity of agricultural enterprises by about 0.067% and 0.159% on average, but the impact is not significant, that is, the increase in the total factor productivity of agribusiness was indeed brought about by the 2013 carbon trading pilot. It can be seen that the pilot carbon trading significantly improved the total factor productivity of agricultural enterprises.

#### 5.3.3. Propensity Score Matching

The coefficients of the interaction term in the previous regression results only represent an “average effect”, that is, the average impact of the carbon trading pilot on the total factor productivity of agricultural enterprises. It is difficult to confirm the real causal relationship between the carbon trading pilot and the total factor productivity of agricultural enterprises. At the same time, whether enterprises are affected by the carbon trading pilot is not random. The change in the total factor productivity of agricultural enterprises is related to enterprise size, R&D investment, and other factors. If the influence of these factors is not excluded, it will inevitably cause sample selection bias. Therefore, this paper adopts the propensity score-matching method to correct the sample selectivity bias to reduce the interference of the experimental results and ensure that the results are more robust. Columns (1)–(3) of Table 5 show the regression results of total factor productivity (TFP) of agribusiness and the implementation of digital finance ($Treat_{}*Time$) after adjacent matching, radius matching, and core matching tests, respectively. The estimated coefficients of the interaction item ($Treat_{}*Time$) are all significantly positive, the pilot policies of carbon emissions trading increased the total factor productivity of agricultural enterprises by about 0.171%, 0.149%, and 0.149% on average, which once again indicates that the carbon trading pilot significantly improves the total factor productivity of agricultural enterprises. These tables show that the coefficient signs and significance levels of the interaction terms are consistent with the results of the previous analysis. This also verifies the basic regression results; that is, the carbon trading pilot is beneficial to improve the total factor productivity of agricultural enterprises and ensures the robustness of the basic regression results.

#### 5.3.4. Explained Variable Replacement

This paper changed the measurement method of the explained variable and tested it again. The results are shown in Column (4) of Table 5. The regression coefficient between the carbon trading pilot and total factor productivity of agricultural enterprises is still significantly positive, the pilot policies of carbon emissions trading increased the total factor productivity of agricultural enterprises by about 0.171%, 0.149%, and 0.165% on average, and the regression result is consistent with the above.

#### 5.3.5. One-Period Lag Explanatory Variable

Considering that the implementation of policies may have a lag effect, this paper conducts basic regression again with the core explanatory variable lagging by one period. The results are shown in Column (5) of Table 5. The regression result is still significantly positive, the pilot policies of carbon emissions trading increased the total factor productivity of agricultural enterprises by about 0.171%, 0.149%, and 0.165% on average, which verifies Hypothesis 1 in this paper.

### 5.4. Mechanism Inspection

The results of the above analysis indicate that the pilot policy of carbon emission trading significantly contributes to the total factor productivity of agricultural enterprises. Then, how does the pilot policy of carbon emission trading affect the total factor productivity of agricultural enterprises? According to the literature, technological innovation affects the level of total factor productivity. Environmental regulation affects total factor productivity through R&D inputs (Wu et al., 2013) [32]. Therefore, this paper chooses green innovation as a mediating variable and uses the mediating effect model to test the mechanism of carbon emission trading rights affecting the total factor productivity of agricultural enterprises. The regression results are shown in Table 6.

Table 6 shows the regression results of the mechanism effect. Columns (1)–(3) list the regression results after adding enterprise green technology innovation, green practical technology innovation, and green invention technology innovation, respectively, into the basic model. The results show that after adding enterprise green technology innovation, the interaction item is significantly positive at the 10% statistical level; for every additional unit of enterprise green technology innovation, the total factor productivity of agricultural enterprises will increase by about 0.011% on average; after adding green practical technology innovation, the interaction item is significantly positive at the 5% statistical level; for each additional unit of green practical technology innovation, the total factor productivity of agricultural enterprises will increase by about 0.019% on average. After adding green invention technology innovation, the coefficients of the interaction terms are all positive but insignificant; for each additional unit of green invention technology innovation, the total factor productivity of agricultural enterprises increases by about 0.024% on average, but the improvement effect is not obvious, indicating that under the pilot policy of carbon emission trading, enterprise green technology innovation significantly promotes the improvement in the total factor productivity of agricultural enterprises, and total factor productivity improvement is achieved by improving the level of green practical technology innovation rather than green invention technology innovation. From the analysis of the mechanism effect model, it can be seen that the implementation of China’s carbon emissions trading makes Chinese agricultural enterprises give more attention to the improvement of their own green innovation level, resulting in an increase in the output of green innovation represented by green patents, especially the improvement of the level of green practical technology innovation. These patent achievements have also been used extensively in practice, which has led to the improvement of the internal production process of Chinese agricultural enterprises, and it also means that a professional energy-saving and emission-reduction technology service industry is being cultivated and formed, creating a channel for carbon emissions trading, which significantly promotes the improvement of agricultural total factor productivity in the pilot areas.

## 6. Further Research

### 6.1. Grouping by Enterprise Size

A large number of studies have shown that the size of an enterprise affects the cost of emission reduction, which in turn has an impact on the environmental protection decision of the enterprise. To investigate whether the difference in the effect of carbon emission trading truly exists under different enterprise scale characteristics, we set up a virtual variable of enterprise scale and divided the sample enterprises into large-scale enterprises and small-scale enterprises. Basic regression was performed on the two groups of samples. The regression results of Columns (1) and (2) in Table 7 show that the carbon emission trading pilot has significantly increased the total factor productivity of large-scale agricultural enterprises by about 0.137% on average, while it has no significant effect on small-scale agricultural enterprises. The possible reason for this situation is that large enterprises not only have the advantage of economies of scale but can also obtain government funding subsidies and low-cost financing by virtue of a good social reputation to make up for the cost of regulatory compliance brought by the carbon emission trading mechanism. At the same time, the strong financial strength of large enterprises can help them resist greater risks and embark on a path of independent transformation with the help of central environmental regulations.

### 6.2. Grouping by Enterprise Ownership

The different natures of property rights of state-owned holding enterprises and private holding enterprises lead to different paths and characteristics in political functions, development goals, market competition and other aspects, and different factors that are considered when making decisions. Therefore, state-owned enterprises and nonstate-owned enterprises may respond differently to the implementation of carbon emission trading policies. We construct the virtual variable of enterprise ownership nature, divided the sample enterprises into state-owned enterprises and nonstate-owned enterprises, and regressed the two samples separately using the differences-in-differences method. The regression results of Columns (3) and (4) in Table 7 show that the carbon emission trading pilot has significant positive effects on total factor productivity in both state-owned and nonstate-owned agricultural enterprises. However, the estimated interaction term coefficient of state-owned agricultural enterprises was 0.097 and significant at the 5% level, and the estimated coefficient of nonstate-owned agricultural enterprises was 0.224 and significant at the 1% level. The impact of the carbon emission trading policy on the total factor productivity of nonstate-owned enterprises is greater than that of state-owned enterprises. This may be because state-owned enterprises assume political functions, and the degree of innovation does not completely depend on their operating capacity. In contrast, private enterprises can adapt to the development of the market in environmental rights and interests in trading policies and independently choose the behaviors conducive to their profit maximization in fierce market competition. Therefore, the carbon trading market has a stronger incentive effect on the green technology innovation of private enterprises.

### 6.3. Grouping by Financial Structure

According to the principle of financial leverage, debt can reduce the total cost of capital and improve the value of enterprises. In addition, when the debt ratio increases, the equity concentration of management becomes larger, and a larger proportion of profits can be obtained in the project. A higher debt ratio also makes firms more willing to invest in riskier projects, such as innovation activities. Enterprises with lower debt levels have a stronger chance of launching research. Therefore, according to the median value of the enterprise asset-liability ratio, this paper divides the sample into high-debt enterprises and low-debt enterprises to test whether the effect of the carbon emission trading policy on the total factor productivity of agricultural enterprises is heterogeneous due to the difference in debt level. It can be seen from the regression results of Columns (5) and (6) in Table 7 that the carbon emission trading pilot significantly increased the total factor productivity of high-debt agricultural enterprises by about 0.137% on average, while it had no significant effect on low-debt agricultural enterprises, most likely because debt, as a signal to the outside world, is often used by management, and a high debt ratio shows management’s confidence in future expectations and, for debtors, a willingness to invest in such companies, thus allowing firms to have sufficient capital for green innovation activities and thus contributing to the total factor productivity of highly indebted agribusinesses.

### 6.4. Grouping by Enterprise Region

The development level of the eastern region and the central and western regions may differ to some extent. The pilot policy of carbon emission trading may have different roles in promoting the total factor productivity of enterprises in different regions. This paper divides the samples into three subsamples: eastern, central and western, according to the different regions where the enterprises are located. As seen from the review results of Columns (1)–(3) in Table 8, the promotion effect of carbon emission trading pilot policies on the total factor productivity of enterprises in the eastern, central and western regions shows a downward trend. The total factor productivity of agricultural enterprises in the eastern, central and western regions increased by about 0.171%, 0.169%, and 0.082%, respectively. Among them, the promotion effect on the total factor productivity of enterprises in the eastern region is the most obvious. The promoting effects on the central and western regions were relatively weak, respectively. The possible reason is that carbon emission trading, as a market-motivated regulatory tool, relies on a unified, open, and orderly competitive market system to play its full role. The eastern region has an active market economy and a developed economic level, so the incentive effect of the carbon emission trading mechanism is more efficient. However, the economic development level of the central and western regions is lagging behind, and their primary goal is to achieve economic development. Therefore, the compliance cost of carbon emission trading will increase the costs and decrease the profits of enterprises in the central and western regions, resulting in a lack of motivation to improve the total factor productivity of enterprises.

### 6.5. Grouping by Government Subsidies

Enterprises with government subsidies usually have more “exogenous resources”, which gives them more opportunities to innovate and invest more, thus gaining a competitive advantage and improving their total factor productivity. To investigate whether the difference in government subsidies in carbon trading is real, we set up a virtual variable for government subsidies by referring to the practices of others and extracted the data of energy saving, emission reduction, green, environmental protection, clean energy, environment, waste gas, carbon emission, and carbon trading fields from the CSMAR by taking the logarithm, and if the value was null, then it was 0. The basic regressions were conducted for the two groups of samples obtained. The results are shown in Columns (4) and (5) of Table 8. The total factor productivity of agricultural enterprises with and without government subsidies is significantly increased under the influence of the carbon trading rights policy. The pilot carbon emissions trading policy significantly increased the total factor productivity of agricultural enterprises without government subsidies by about 0.150% on average, while the total factor productivity of agricultural enterprises with government subsidies increased by about 1.973% on average. The estimated coefficient of nongovernment subsidies is 0.150 and that of nongovernment subsidies is 1.973, which is greater than that of nongovernment subsidy enterprises. The possible reason is that government subsidies can provide enterprises with external resources to deal with environmental uncertainties, enabling enterprises to turn opportunities brought by environmental changes into an innovation impetus, forming a synergistic effect with innovation input and promoting the improvement of enterprise internationalization performance.

## 7. Conclusions and Policy Suggestions

### 7.1. Conclusions

The carbon emission trading mechanism is an important policy tool in using market mechanisms to promote corporate carbon emission reduction, an important institutional innovation to promote low-carbon and green development, and an important institutional arrangement to promote the “carbon peak in 2030 and carbon neutral in 2060”. Therefore, this paper empirically examines the impact of carbon emission trading rights on the total factor productivity of agricultural enterprises using a double-difference model and a mediating effect model using data from listed agricultural companies in Shanghai and Shenzhen A-shares and GEM from 2010 to 2020 and investigates the mechanism of its impact in depth. The main conclusions are as follows.

(1) Carbon emission trading rights significantly increase the total factor productivity of agricultural enterprises.
(2) Green innovation plays a mechanistic role in the influence of carbon emission trading rights on the total factor productivity of agricultural enterprises.
(3) Heterogeneity analysis shows that the improvement effect of carbon emission trading rights on the total factor productivity of agricultural enterprises mainly exists in large-scale, nonstate-owned enterprises; enterprises with high debt levels; enterprises in the eastern region; and enterprises with government subsidies.

Limitations. First, due to the serious lack of relevant data before 2010, in order to ensure the accuracy of the results of the article, the data used in this empirical test starts from 2010. Second, China’s current carbon emission trading is still in the pilot stage, and the transaction price is subject to excessive government control. At present, there is no continuous and large amount of transaction data, and it is impossible to conduct effective research on the entire market. Third, the total factor productivity of agricultural enterprises is a complex project. This paper only conducts empirical tests from the perspective of the green innovation effect, and other potential impact mechanisms and paths still need to be identified. Subsequent research can include all possible influencing factors, continue to improve the intermediate action mechanism of carbon emission trading policy on the total factor productivity of agricultural enterprises, and clarify their respective weights in the driving of total factor productivity of agricultural enterprises.

### 7.2. Policy Suggestions

Based on the above conclusions, we can give the following recommendations.

(1) We will improve and optimize China’s rules and regulations on carbon emission trading, ensure that the transactions in the trading market are carried out legally and in an orderly manner, play a role in promoting green innovation in agricultural enterprises and promoting total factor productivity, and help agricultural enterprises transform and upgrade. At the same time, individuals trading in the carbon emission trading market should consciously abide by the market order, create a good trading market environment, and further promote the total factor productivity of agricultural enterprises.
(2) Green innovation is an effective way to realize the leap-forward of total factor productivity of agricultural enterprises and the long-term goal of continuously deepening the carbon trading mechanism. The government should strengthen green innovation compensation, enhance the capacity of independent research and development of enterprises and cooperative innovation, optimize the structure of green industry, and improve the support of environmental protection standards and management norms, inducing enterprises to complete the transformation of “innovation compensation”. At the same time, we should further improve the incentive system of government rewards and punishments and green innovation, give full play to the incentive effect of government reward and punishment, fully encourage enterprises to engage in green technology innovation, and guide enterprises to achieve improvements in profitability in the stage of “following the cost”.
(3) According to the current situation and characteristics of agricultural enterprises with different scales, ownership properties, regions, and debt levels in China, differentiated regulation strategies should be implemented to avoid the adoption of “one-size-fits-all” regulation orders. At the same time, different types of enterprises should be treated equally to create a favorable competitive environment and to promote environmental regulatory instruments to maximize policy effects.
(4) Enterprises are encouraged to properly respond to carbon emission trading policies through R&D and innovation to achieve “decoupling” between high production capacity and high carbon emissions as soon as possible. At the same time, we should speed up the establishment of a national carbon emissions trading system to avoid transregional carbon emissions transfer, facilitate China’s transition to a zero-carbon economy, and make positive contributions to leading global climate governance.

## Figures and Tables

**Table 1:** Definition of each variable.

Variable Symbol	Variable Name	Variable Description
TFP	total factor productivity of agribusiness	by CD production function method, OP method, LP method
Size	Enterprise size	Ln (total assets at the end of the period)
Roa	Return on Assets	Net profit/Total assets
Lev	Assets and liabilities	Total liabilities at the end of the period/total assets at the end of the period
Agencost	agency cost	Administrative expenses/main business income
Cflow	cash flow from operating activities	Net cash flow from operating activities/total assets at the end of the period
Capital	factor density	Ln (real net fixed assets per capita)
Top1	Shareholding ratio of the largest shareholder	Shareholding ratio of the largest shareholder
Patent	Enterprise green innovation	The total number of green patent applications
Invent	Enterprises invent green innovation	Number of green invention patent applications
Actual	Enterprise practical green innovation	Green utility model patent application

**Table 2:** Descriptive statistics of the main variables.

Variable	N	Mean	P50	Sd	Min	Max
TFP (OP)	1340	6.600	6.547	0.750	4.905	8.363
TFP (LP)	1340	9.081	8.985	1.016	6.733	11.670
Patent	1340	1.726	0.000	4.519	0.000	30.000
Invent	1340	1.058	0.000	3.033	0.000	20.000
Actual	1340	0.590	0.000	1.651	0.000	11.000
TreatPost	1340	0.219	0.000	0.414	0.000	1.000
Age	1340	18.880	19.000	5.042	7.000	31.000
Roa	1340	0.049	0.042	0.076	−0.243	0.258
Size	1340	7.916	7.793	1.211	5.112	11.020
Lev	1340	0.387	0.370	0.184	0.043	0.900
Agencost	1340	0.079	0.063	0.063	0.014	0.439
Cflow	1340	0.069	0.065	0.087	−0.183	0.319
Capital	1340	0.266	0.247	0.137	0.017	0.609
Subsidy	1340	11.900	15.290	6.921	0.000	19.620
Top1	1219	36.270	35.880	14.680	9.270	70.320
GDP	1340	10.920	10.890	0.470	9.889	12.010

**Table 3:** Basic regression results.

Variable	TFP	TFP	TFP
	(1)	(2)	(3)
Treat * Time	0.316 ***	0.149 ***	0.181 ***
	(7.09)	(4.87)	(5.47)
Size		0.325 ***	0.551 ***
		(15.80)	(41.09)
Roa		0.806 ***	2.101 ***
		(5.51)	(7.91)
Lev		0.480 ***	0.851 ***
		(6.37)	(9.74)
Agencost		−3.503 ***	−4.456 ***
		(−19.00)	(−18.66)
Cflow		0.450 ***	0.654 ***
		(4.28)	(3.19)
Capital		−1.387 ***	−1.795 ***
		(−12.98)	(−16.30)
Top1		−0.009 ***	−0.002 **
		(−6.67)	(−2.14)
Constant	9.015 ***	7.212 ***	5.114 ***
	(650.79)	(42.47)	(48.75)
Year fixed effects	control	control	not controlled
Individual fixed effects	control	control	not controlled
Observations	1330	1210	1219
R-squared	0.890	0.960	0.976
F	206.8	206.8	206.8

Note: *, **, *** indicate significance at the 10%, 5%, and 1% levels, respectively.

**Table 4:** Robustness check.

Variable	TFP		
	(1) Parallel Trend Test	(2) Test 2012	(3) Test 2011
Pre3	−0.009		
	(−0.08)		
Pre2	0.135		
	(1.33)		
Pre1	0.060		
	(0.61)		
Current	0.121		
	(1.23)		
After1	0.207 **		
	(2.12)		
After2	0.220 **		
	(2.37)		
After3	0.168 *		
	(1.90)		
After4	0.175 **		
	(2.05)		
After5	0.241 ***		
	(2.85)		
After6	0.209 **		
	(2.34)		
Placebox1		0.067	0.159
		(0.57)	(1.21)
Size	0.577 ***	0.262 ***	0.196 ***
	(38.45)	(9.14)	(5.98)
Roa	2.282 ***	1.061 ***	2.326 ***
	(8.71)	(3.92)	(5.21)
Lev	0.934 ***	0.325 **	1.134 ***
	(9.71)	(2.03)	(4.46)
Agencost	−1.021 ***	−4.752 ***	−6.979 ***
	(−8.63)	(−10.74)	(−9.05)
Cflow	0.599 ***	0.181	0.302
	(2.84)	(1.05)	(1.35)
Capital	−1.836 ***	−0.775 ***	−0.768 ***
	(−14.81)	(−3.72)	(−2.97)
Top1	−0.003 **	−0.001	−0.002
	(−2.37)	(−0.23)	(−0.59)
Constant	4.634 ***	7.282 ***	7.578 ***
	(41.37)	(30.00)	(27.50)
Year fixed effect	Control	Control	Control
Individual fixed effect	Control	Control	Control
Observations	1219	308	196
R-squared	0.717	0.001	0.001
F	178.6	0.982	0.982

Note: *, **, *** indicate significance at the 10%, 5%, and 1% levels, respectively.

**Table 5:** Robustness check.

Variable	TFP				
	(1)	(2)	(3)	(4)	(5)
Treat * Time	0.171 ***	0.149 ***	0.149 ***	0.165 ***	0.179 ***
	(3.40)	(4.87)	(4.88)	(4.57)	(5.20)
Size	0.340 ***	0.325 ***	0.323 ***	−0.054 **	0.339 ***
	(9.75)	(15.80)	(15.68)	(−2.40)	(13.60)
Roa	0.556 **	0.806 ***	0.770 ***	1.110 ***	0.961 ***
	(2.36)	(5.51)	(5.18)	(7.73)	(6.46)
Lev	0.440 ***	0.480 ***	0.470 ***	0.677 ***	0.551 ***
	(3.33)	(6.37)	(6.21)	(7.95)	(6.22)
Agencost	−3.292 ***	−3.503 ***	−3.521 ***	−0.885 ***	−0.813 ***
	(−10.84)	(−19.00)	(−19.05)	(−12.88)	(−12.08)
Cflow	0.510 ***	0.450 ***	0.472 ***	0.472 ***	0.468 ***
	(2.73)	(4.28)	(4.44)	(4.28)	(4.10)
Capital	−1.410 ***	−1.387 ***	−1.381 ***	−1.428 ***	−1.676 ***
	(−8.41)	(−12.98)	(−12.93)	(−11.50)	(−13.27)
Top1	−0.007 ***	−0.009 ***	−0.009 ***	−0.011 ***	−0.010 ***
	(−3.06)	(−6.67)	(−6.68)	(−6.74)	(−5.87)
Constant	7.022 ***	7.212 ***	7.232 ***	7.507 ***	6.964 ***
	(23.54)	(42.47)	(42.44)	(41.09)	(34.87)
Time effect	control	control	control	control	control
Individual effect	control	control	control	control	control
Observations	479	1210	1209	1210	1109
R-squared	0.972	0.960	0.960	0.900	0.950
F	205.3	205.3	205.3	84.33	30.61

Note: *, **, *** indicate significance at the 10%, 5%, and 1% levels, respectively.

**Table 6:** Mechanism inspection.

Variable	TFP		
	(1)	(2)	(3)
Treat * Time * Patent/Invent/Actual	0.011 *	0.019 **	0.024
	(1.92)	(2.03)	(1.54)
Treat * Time	0.112 ***	0.114 ***	0.114 ***
	(3.19)	(3.30)	(3.20)
Size	0.543 ***	0.543 ***	0.545 ***
	(39.57)	(39.72)	(39.83)
Roa	2.181 ***	2.178 ***	2.186 ***
	(8.16)	(8.14)	(8.16)
Lev	0.889 ***	0.888 ***	0.886 ***
	(10.13)	(10.13)	(10.10)
Agencost	−4.455 ***	−4.454 ***	−4.455 ***
	(−18.55)	(−18.55)	(−18.54)
Cflow	0.613 ***	0.612 ***	0.608 ***
	(2.97)	(2.97)	(2.95)
Capital	−1.779 ***	−1.779 ***	−1.783 ***
	(−16.16)	(−16.16)	(−16.19)
Top1	−0.002 *	−0.002 *	−0.002 *
	(−1.87)	(−1.84)	(−1.93)
Constant	5.159 ***	5.156 ***	5.150 ***
	(48.47)	(48.61)	(48.42)
Year fixed effect	Control	Control	Control
Individual fixed effect	Control	Control	Control
Observations	1219	1219	1219
R-squared	0.779	0.779	0.779
F	460.3	460.5	459.6

Note: *, **, *** indicate significance at the 10%, 5%, and 1% levels, respectively.

**Table 7:** The first set of tests for heterogeneity.

Variable	TFP					
	(1) Large Scale	(2) Small Scale	(3) State Owned	(4) Nonstate Owned	(5) High Debt	(6) Low Debt
Treat * Time	0.137 ***	0.159	0.097 **	0.224 ***	0.227 ***	0.032
	(3.75)	(0.44)	(2.25)	(4.46)	(4.13)	(0.73)
Size	0.322 ***	0.389	0.245 ***	0.361 ***	0.269 ***	0.480 ***
	(14.58)	(0.57)	(7.63)	(12.89)	(8.69)	(13.65)
Roa	1.049 ***	1.763	0.842 ***	0.975 ***	0.453 **	1.505 ***
	(7.48)	(0.50)	(3.94)	(5.36)	(2.03)	(8.00)
Lev	0.627 ***	0.968	0.205 *	0.801 ***	0.362 **	1.305 ***
	(7.49)	(0.68)	(1.75)	(7.27)	(2.42)	(7.49)
Agencost	−0.749 ***	−14.822	−3.700 ***	−0.577 ***	−1.039 ***	−0.231 ***
	(−11.19)	(−0.95)	(−14.33)	(−8.44)	(−9.72)	(−2.65)
Cflow	0.450 ***	0.346	0.313 **	0.520 ***	0.316 **	0.389 ***
	(4.16)	(0.28)	(2.33)	(3.37)	(2.11)	(2.61)
Capital	−1.641 ***	−1.206	−1.592 ***	−1.518 ***	−1.923 ***	−1.404 ***
	(−13.47)	(−0.72)	(−10.01)	(−9.34)	(−8.90)	(−9.68)
Top1	−0.009 ***	0.015	−0.011 ***	−0.006 ***	−0.010 ***	−0.010 ***
	(−5.76)	(0.99)	(−4.03)	(−3.12)	(−4.19)	(−4.03)
Constant	7.020 ***	6.142	8.139 ***	6.467 ***	7.806 ***	5.484 ***
	(39.18)	(1.14)	(29.33)	(29.73)	(29.16)	(19.64)
Time effect	control	control	control	control	control	control
Individual effect	control	control	control	control	control	control
Observations	602	605	547	663	590	599
R-squared	0.948	0.998	0.956	0.953	0.946	0.965
F	2.485	2.485	100.4	100.4	86.71	86.71

Note: *, **, *** indicate significance at the 10%, 5%, and 1% levels, respectively.

**Table 8:** The first set of tests for heterogeneity.

Variables	TFP				
	(1) East	(2) Central	(3) West	(4) Government Subsidy	(5) Anarchy Subsidy
Treat * Time	0.171 ***	0.169	0.082	1.973 ***	0.150 ***
	(5.02)	(0.88)	(0.62)	(4.12)	(7.15)
Size	0.395 ***	0.333 ***	0.256 ***	0.296 ***	0.363 ***
	(12.44)	(6.73)	(6.50)	(11.88)	(5.07)
Roa	1.228 ***	0.573	0.543 *	1.246 ***	0.706 ***
	(6.85)	(1.64)	(1.77)	(7.70)	(3.00)
Lev	0.559 ***	1.101 ***	0.516 ***	0.680 ***	0.693 ***
	(5.45)	(5.61)	(2.91)	(7.33)	(3.82)
Agencost	−1.008 ***	−0.384 ***	−2.677 ***	−0.538 ***	−1.898 ***
	(−11.02)	(−3.58)	(−7.72)	(−7.22)	(−11.66)
Cflow	−0.036	0.303	0.913 ***	0.461 ***	0.714 ***
	(−0.25)	(1.22)	(4.65)	(3.95)	(3.39)
Capital	−1.491 ***	−1.727 ***	−1.580 ***	−1.501 ***	−1.417 ***
	(−10.35)	(−4.96)	(−6.72)	(−10.72)	(−6.24)
Top1	−0.007 ***	−0.012 ***	−0.010 ***	−0.009 ***	−0.010 ***
	(−3.63)	(−3.45)	(−3.05)	(−4.48)	(−2.69)
Constant	6.380 ***	6.928 ***	7.760 ***	7.096 ***	6.279 ***
	(25.85)	(18.60)	(21.04)	(34.81)	(10.36)
Time effect	control	control	control	control	control
Individual effect	control	control	control	control	control
Observations	642	266	302	907	266
R-squared	0.955	0.919	0.963	0.953	0.987
F	41.71	41.71	41.71	36.89	36.89

Note: *, *** indicate significance at the 10%, and 1% levels, respectively.

## Data Availability

The data presented in this study are openly available in National Bureau of Statistics, reference numbers 978-7-5037-9120-8, 978-7-5037-8770-6, 978-7-5037-8432-3, 978-7-5037-8082-0, 978-7-5037-7706-6, 978-7-5037-7350-1, 978-7-5037-7019-7, and 978-7-5037-6754-8.
